# Editorial: The impact of COVID-19 on vulnerable populations

**DOI:** 10.3389/fpubh.2023.1267723

**Published:** 2023-09-08

**Authors:** Echu Liu, Caress A. Dean, Keith T. Elder

**Affiliations:** ^1^Department of Health Management and Policy, College for Public Health and Social Justice, Saint Louis University, Saint Louis, MO, United States; ^2^School of Health Sciences, Oakland University, Rochester, MI, United States; ^3^Office of the Provost, Mississippi College, Clinton, MS, United States

**Keywords:** COVID-19, influence, pandemic, populations, vulnerable

## Introduction

The COVID-19 pandemic has been a harsh reminder of the stark health disparities in our societies. Vulnerable populations, including older adults, women, low-income communities, racial and ethnic minorities, individuals with underlying health conditions, and people without housing, have faced disproportionate challenges during this global health crisis. In this editorial, we examine *The impact of COVID-19 on vulnerable populations* from a public health perspective, highlighting the need for targeted interventions and a more equitable approach to safeguarding public health. The following section lists some critical aspects of the impact and summarizes the findings of the studies on the Research Topic.

## The impact of COVID-19 on vulnerable populations

1. Higher Infection and Mortality Rates:
  Vulnerable populations, such as older adults and individuals with underlying health conditions (e.g., diabetes, heart disease, and respiratory illnesses), lower income, or immunocompromised bodies, experienced higher COVID-19 infection, and mortality rates. These groups often have weakened immune systems or pre-existing health conditions that make them more susceptible to severe outcomes if infected. In some settings, women may have been overrepresented in healthcare and frontline jobs, putting them at higher risk of exposure to the virus. They may also have played a significant role in caregiving, professionally and within their own families, potentially increasing their risk of exposure.
2. Acevedo-Sánchez et al.'s findings show that age significantly predicted one's probability of being infected with COVID-19 during the first pandemic wave in Mexico. For example, older Mexicans with diabetes, hypertension, or obesity were more likely to be infected than older Mexicans without these health issues. Their study also shows that chronic illness was significantly associated with COVID-19 case mortality rates for the same period. Another critical finding is that female Mexicans with metabolic or cardiovascular diseases had a higher mortality rate due to COVID-19 than their counterparts. The analysis by Gerken et al. demonstrates that age was positively associated with COVID-19 case fatalities, whereas income was negatively associated with COVID-19 case fatalities in the United States. Wu and Qian reviewed data collected from March 2020 to February 2022 by Canada's Public Health Agency to study the gender difference in infection rate during the peak of the COVID-19 pandemic. Their work shows that women had a steadily higher infection rate than men during the study period. According to the empirical evidence in this research, this gender disparity can be explained by women's higher share of care work during the pandemic. Santa-Ramírez et al. analyzed data collected from 2,889 participants of a 2020 population-based survey conducted in Geneva, concluding that individuals with financial hardship had higher odds of being infected with COVID-19.
3. Health Care Access Disparities:
  Access to quality healthcare was a significant challenge for vulnerable populations during the pandemic. For instance, many disabled individuals, a considerable portion of the vulnerable group, rely on public transportation or specialized services for mobility. During the pandemic, lockdowns, reduced services, and safety concerns might limit their ability to access transportation, making it difficult to travel to essential places such as medical appointments. Therefore, their ability to access appropriate healthcare can be negatively impacted. In addition, individuals with underlying health conditions may have been less physically active, particularly outside their homes, due to the fear of contracting the virus. This reduction in physical activity is often associated with adverse health consequences.
  Sohn et al. analyzed matched samples from the 2015 to 2020 Korean National Health Insurance's claim records to determine the influence of the pandemic on disabled individuals' healthcare use. The difference-in-differences estimates calculated in this study indicate that the pandemic significantly reduced disabled Koreans' use of medical care and that the degree of decline is positively associated with the severity of the disability. Additionally, this study shows that those with a physical disability experienced the most significant reduction compared to individuals with other disabilities. A qualitative survey by Krczal and Hyll indicates that the COVID-19 pandemic deteriorated the pattern of physical activities of Austrians with cardiovascular diseases in its initial stage. Despite all odds, this pattern did improve as the pandemic progressed.
4. Health Communication Challenges:
  Public health communication efforts occasionally struggle to reach vulnerable populations effectively. Language barriers, literacy issues, and limited internet access hindered people's ability to receive accurate information about COVID-19, preventive measures, and vaccination. Significantly, the shift to online health education and virtual healthcare services during the pandemic highlighted the digital divide among vulnerable populations. Lack of access to reliable internet connections, devices, and digital literacy skills also hindered their ability to participate fully in remote activities and access essential information, potentially exacerbating existing inequities and limiting their opportunities for support and engagement. Ritcher and Heidinger's findings suggest that older Austrians in poverty were less likely to use the internet than younger and financially stable Austrians during the pandemic. Wang et al. analyzed the 2020 China Family Panel Studies cross-sectional data. They concluded that the intensity of internet use is associated with the quality of life among chronically ill Chinese during the pandemic.
5. Mental Health and Wellbeing:
  The pandemic's toll on mental health has been profound, particularly among vulnerable populations. The stress and anxiety, economic hardships, and social isolation related to the virus have affected these communities disproportionately. However, mental health support and resources may not have been readily available or culturally appropriate, exacerbating the strain on their wellbeing.
  Schippers et al.'s findings show that the public health measures adopted to slow the spread of COVID-19 disturbed and reduced the social connections of older adults during the pandemic. This study also shows that the public health measures worsened the pre-existing disparities in the social relationships among racial and ethnic groups. Islam et al. analyzed data from three waves from the US COVID-19 Impact Survey to identify characteristics associated with financial hardship and to evaluate the associations of final difficulty with mental health symptoms among cancer survivors during the pandemic. According to this study, minorities, younger adults, and cancer survivors with low socioeconomic status had a higher chance of financial hardship during the COVID-19 crisis, resulting in anxiety, depression, and hopelessness.
6. Social and Economic Consequences:
  The socioeconomic consequences of the pandemic have been particularly devastating for vulnerable populations. Many lost their jobs or faced reduced working hours, leading to financial instability and increased vulnerability to food insecurity, homelessness, and other hardships. Dean et al. analyzed data from three US COVID-19 Impact Survey waves. They found that approximately one-third of the study sample with chronic illness experienced food shortages in the initial phase of the COVID-19 pandemic. The results from this study suggest that chronically ill Americans with lower socioeconomic status, such as those with lower income or less education, have a higher risk of food shortage.
7. Vaccination Inequities:
  Vaccine distribution and accessibility have not been equitable, leading to lower vaccination rates in vulnerable communities. Issues such as vaccine hesitancy, limited access to vaccination sites, and mistrust of healthcare systems have contributed to disparities in vaccination coverage. An analysis by Ritcher and Heidinger shows that although older Austrians in poverty were significantly more likely to refuse COVID-19 vaccination, they adhered to other public health measures.
8. Systemic Inequities and Structural Racism:
  COVID-19 exposed and amplified systemic inequities and structural racism, perpetuating disparities among vulnerable populations. In some regions, women faced barriers to accessing healthcare, leading to potential negative consequences for women's health during the pandemic. Moreover, racial and ethnic minorities, indigenous communities, immigrants, and refugees experienced higher infection rates, inadequate healthcare, and discriminatory treatment.
  Samanta et al.'s findings suggest that women living in Tamil Nadu, India, had a lower rate of detected cases than men. In 2020, Oliveira Martins et al. surveyed a cohort of 410 households in Amadora Municipality, Lisbon Region in Spain, to examine COVID-19's socioeconomic impact on immigrants, who remain among the most vulnerable and neglected members of many societies. Their statistical analysis shows that the COVID-19 pandemic exacerbated the pre-existing socioeconomic inequalities between immigrants and non-immigrants in the area. Compared to natives, this study found that the likelihood of immigrants in the region losing jobs and being laid off was higher during the pandemic. Furthermore, immigrants' possibility of facing financial hardship, such as difficulties buying food and hygiene products and paying bills, was also higher. Plümecke et al. analyzed Swiss mortality statistics and concluded that non-Swiss citizens had higher death rate increases than Swiss citizens during the first two waves of the pandemic. This finding suggests that the Swiss healthcare system does not protect all citizens equally in a public health crisis like the COVID-19 pandemic. The pandemic has laid bare the urgent need to address the underlying social determinants of health and work and build toward a more equitable society.

## Call to action

Addressing *The impact of COVID-19 on vulnerable populations* requires a multifaceted and targeted approach. Governments, healthcare systems, and community organizations must prioritize equitable vaccine distribution, improve access to healthcare services, and ensure culturally sensitive and linguistically appropriate information dissemination. Officials should strengthen social safety nets to support those facing economic hardships, intensify efforts to bridge the digital divide and ensure mental health services are accessible and affordable for everyone. Addressing systemic inequities and racism is crucial for long-term change.

## Conclusion

Ultimately, it is essential to approach the pandemic response with an equity lens to ensure that vulnerable populations are not left behind and that public health efforts are inclusive and effective for all segments of society. The COVID-19 pandemic has further exposed the vulnerabilities and disparities experienced by marginalized populations. Schippers et al. demonstrated that the crisis hit vulnerable people hardest. Therefore, we must focus on protecting and supporting vulnerable communities as we navigate the recovery phase and continue to build a more resilient and inclusive society that leaves no one behind by addressing each person's specific challenges and working toward equitable solutions. We can emerge stronger from this crisis and ensure a more equitable future through collective action and commitment to social justice.

## Author contributions

EL: Writing—original draft. CD: Writing—review and editing. KE: Writing—review and editing.

